# Methotrexate used in combination with aminolaevulinic acid for photodynamic killing of prostate cancer cells

**DOI:** 10.1038/sj.bjc.6603273

**Published:** 2006-07-25

**Authors:** A K Sinha, S Anand, B J Ortel, Y Chang, Z Mai, T Hasan, E V Maytin

**Affiliations:** 1 Department of Dermatology, Wellman Center for Photomedicine, Harvard Medical School, Boston, MA 02114, USA; 2 Department of Dermatology, Cleveland Clinic Foundation, Cleveland, OH 44195, USA; 3 Department of Biomedical Engineering, Cleveland Clinic Foundation, Cleveland, OH 44195, USA; 4 Department of Medicine, Massachusetts General Hospital, Harvard Medical School, Boston, MA 02114, USA

**Keywords:** chemotherapy, phototherapy, cellular differentiation, porphyrin

## Abstract

Photodynamic therapy (PDT) using 5-aminolaevulinic acid (ALA) to drive production of an intracellular photosensitiser, protoporphyrin IX (PpIX), is a promising cancer treatment. However, ALA-PDT is still suboptimal for thick or refractory tumours. Searching for new approaches, we tested a known inducer of cellular differentiation, methotrexate (MTX), in combination with ALA-PDT in LNCaP cells. Methotrexate alone promoted growth arrest, differentiation, and apoptosis. Methotrexate pretreatment (1 mg l^−1^, 72 h) followed by ALA (0.3 mM, 4 h) resulted in a three-fold increase in intracellular PpIX, by biochemical and confocal analyses. After exposure to 512 nm light, killing was significantly enhanced in MTX-preconditioned cells. The reverse order of treatments, ALA-PDT followed by MTX, yielded no enhancement. Methotrexate caused a similar relative increase in PpIX, whether cells were incubated with ALA, methyl-ALA, or hexyl-ALA, arguing against a major effect upon ALA transport. Searching for an effect among porphyrin synthetic enzymes, we found that coproporphyrinogen oxidase (CPO) was increased three-fold by MTX at the mRNA and protein levels. Transfection of LNCaP cells with a CPO-expressing vector stimulated the accumulation of PpIX. Our data suggest that MTX, when used to modulate intracellular production of endogenous PpIX, may provide a new combination PDT approach for certain cancers.

Photodynamic therapy (PDT) using 5-aminolaevulinic acid (ALA) as a prodrug that is converted to an intracellular photosensitiser, protoporphyrin IX (PpIX), represents an increasingly accepted treatment for superficial epithelial neoplasias (Kennedy *et al*, 1990; Marmur *et al*, 2004) and for diagnostic imaging of internal malignancies at locations where light can be delivered through an optical fibre (Kennedy *et al*, 1996; Eker *et al*, 1999; Landry *et al*, 2003; Schmidbauer *et al*, 2004). However, ALA-mediated PDT (ALA-PDT) remains suboptimal for the treatment of deeper tumours, such as nodular basal cell and squamous cell carcinoma of the skin, because recurrence rates after single treatments are unacceptably high as compared to the current standard of care, surgical excision (Marmur *et al*, 2004). For internal malignancies in organs such as the bladder, GU tract, and oesophagus, ALA-PDT has been used for palliative treatment of these cancers (Kennedy *et al*, 1996; Waidelich *et al*, 2001; Friesen *et al*, 2002). However, ALA-PDT in its current form seldom achieves a definitive cure of deep or refractory tumours in any location. Clearly, new approaches are needed, including ways to increase the clinical responsiveness of tumour cells to ALA-PDT.

Methotrexate (MTX) is a widely used and highly successful anticancer agent. A structural analogue of folic acid and a potent inhibitor of dihydrofolate reductase, MTX interferes with the synthesis of thymidylate and purine nucleotides (Hatse *et al*, 1999), and blocks DNA synthesis, thereby inhibiting tumour growth. However, another important property of MTX is its ability to trigger cellular differentiation in a number of tumour cells, including human and rat choriocarcinoma cells (Friedman and Skehan, 1979; Hatse *et al*, 1998), human promyelocytic HL-60 cells (Bodner *et al*, 1981), human neuroblastoma LA-N-1 cells (Ross *et al*, 1995), as well as normal human keratinocytes (Schwartz *et al*, 1992). This differentiation-promoting property of MTX is of particular interest to us. Malignant cells typically display an uncoupling of the balance between cellular proliferation and differentiation, in which cellular proliferation is favoured owing to a block in maturation (Marks and Rifkind, 1991). However, in certain cases, differentiation-inducing agents can reactivate a tumour cell's differentiation programme and bypass this defective growth regulation, forcing cells into a more differentiated state that has a more favourable clinical prognosis. One major clinical example of this is the use of all-*trans* retinoic acid for differentiation therapy of acute promyelocytic leukaemia (Douer, 2000). Another well-known example is the successful treatment of highly aggressive human choriocarcinoma tumours using MTX, which was shown to induce cytodifferentiation in human and rat choriocarcinoma cells in culture (Berkowitz *et al*, 1986).

Because MTX can induce cellular differentiation in some types of tumour cells, and because we have recently shown that agents capable of inducing cellular differentiation will also cause an elevation in the level of PpIX photosensitiser in epithelial cells of the skin (Ortel *et al*, 1998) and prostate (Ortel *et al*, 2002), we became interested in the notion of combination therapy involving MTX and ALA-PDT. Specifically, we reasoned that if MTX could promote differentiation and PpIX accumulation in carcinoma cells, then MTX might serve as a means to augment the efficacy of ALA-PDT for tumour cell killing. The data described in this paper, obtained using a carcinoma cell line, suggest that MTX may indeed be a useful adjunct to ALA-PDT when administered before the phototherapy.

## MATERIALS AND METHODS

### Source of reagents

ALA and MTX were from Sigma-Aldrich (St Louis, MO, USA). Methyl-ALA and hexyl-ALA were obtained from ORGANIX (Department of Biological Sciences, University of Essex, Wivenhoe Park, Colchester, UK). R1881 was obtained from Perkin-Elmer Life Sciences (Boston, MA, USA).

### Culture of LNCaP cells

LNCaP human prostate carcinoma cells were obtained from American Type Culture Collection (ATCC; Manassas, VA, USA) and cultured at 37°C in a humidified CO_2_ incubator in Rosewell Park Memorial Institute (RPMI) 1640 medium with L-glutamine (Mediatech Inc., Herndon, VA, USA) supplemented with 10% foetal bovine serum (Invitrogen Corp., Carlsbad, CA, USA) and 1% penicillin–streptomycin and 10 mM HEPES buffer. To maintain reproducible behaviour of the LNCaP cells, we found it important to keep the cells subconfluent. The original vial of cells was thawed and plated in the medium in a 100 mm dish, and at 24 h, the medium was replaced and left on for another 3–4 days. When nearly confluent, the cells were released with 0.25% trypsin (noting under the microscope when cells had just detached, usually <3 min), and were replated in RPMI medium at a 1 : 3 dilution. Subsequently, cells were passaged at 1 : 3 dilution every 3–4 days, always before they reached full confluence.

### Measurement of proliferation, differentiation and apoptosis

Control LNCaP cells were plated in 35 mm dishes at an initial density of ∼100 000 cells dish^−1^, and after 72 h of growth, were trypsin-released and counted. Methotrexate-treated dishes were plated at a higher initial density of ∼250 000 cells dish^−1^, to give a final cell density that was roughly equal to the controls after 72 h. Proliferation was estimated as the ratio (percentage) of the final trypsinised cell number divided by the number of cells plated. Growth arrest was assessed by upregulation of p27/kip-1, and differentiation by upregulation of E-cadherin in MTX-treated LNCaP cells, as described (Ortel *et al*, 2002).

Apoptosis was measured by a fluorescein isothiocyanate (FITC)-annexin V/propidum iodide assay (Vybrant Apoptosis Assay, Molecular Probes Inc., Eugene, OR, USA). Supernatants from each culture dish were collected in 15 ml Falcon tubes, cells were trypsin-released (0.5 ml of trypsin) and pooled with the respective supernatant, and cells pelleted for 10 min at 1000 r.p.m. in a refrigerated centrifuge (20°C). The supernatant was discarded, the cell pellet was washed by gentle resuspension in phosphate-buffered saline, repelleted, and the cell pellet was resuspended in 100 *μ*l of 1 : 5 diluted buffer supplied in the kit. In a fresh microcentrifuge tube, 20 *μ*l of the cell suspension was mixed with 80 *μ*l of 1 : 5 diluted buffer, then 5 *μ*l of annexin-FITC and 1 *μ*l of 1 : 10 diluted propidium iodide (supplied in the kit) was added in each tube. After 15 min at room temperature, 400 *μ*l of 1 : 5 diluted buffer was added and the cell suspension was transferred to a fluorescent-activated cell sorting (FACS) tube on ice (Becton Dickinson Labware, Bedford, MA, USA) and measured by fluorescent-activated cell analysis (FACSCalibur, Becton Dickinson, CA, USA).

### Pretreatment of cells with MTX or R1881, and measurement of PpIX in cell lysates

In all cases, cells were plated at ∼80–100k for untreated dishes and ∼180–250k for MTX-treated dishes, in order to yield an approximately equal cell density at the end of the experiment. Methotrexate (1 mg l^−1^ in media) was added on the second day after plating and incubated for an additional 72 h, after which the medium was aspirated and ALA in fresh medium was added. In the experiment shown in Figure 3A, the androgen analogue R1881 (0.1 *μ*M) was added for 72 h to some of the dishes. For the last 4 h of all experiments, ALA (at 0.3 mM concentration, unless otherwise noted) was added to the culture medium, the cells trypsin-released, and final cell counts were determined with a haemocytometer. The cells were lysed and vortexed in 2 ml of 1% sodium dodecyl sulphate in 0.1 N NaOH. The amount of PpIX per cell was determined as follows. An aliquot of cell lysate was measured in a scanning spectrophotometer (Horiba Instruments SA Inc., Jobin Yvon/Spex Division, Edison, NJ, USA) and the peak area between 580 and 720 nm was determined. The PpIX concentration of each sample was calculated by comparison to a calibration curve constructed from measuring PpIX standards, run on the same day. Based upon the known volume of the lysate and the cell count before lysis, the PpIX concentration was converted to total PpIX (in pmol) per 1 million cells.

### Fluorescence analysis of PpIX in living cells

Protoporphyrin IX-specific fluorescence in living LNCaP cells was analysed by fluorescence microscopy on a confocal laser-scanning microscope (Leica Microscopy Systems GmbH, Wetzlar, Germany). LNCaP cells were plated on microscope coverslips in 35 mm dishes at 50 000 cells dish^−1^, then 24 h later the culture media were replaced either with media alone or with media containing MTX (1 mg l^−1^). The cells were cultured for an additional 72 h, then ALA (0.3 mM) was added to the wells for a final 4 h of incubation. Protoporphyrin IX-specific fluorescence in the living cells was analysed on the confocal microscope. Using excitation at 488 nm, images were collected in the red channel, through a 590 nm long-pass filter.

### Transient transfection of LNCaP cells for analysis of PpIX fluorescence

LNCaP cells (3 × 10^5^ cells dish^−1^) were plated on a 22 × 22 mm, No. 1 cover glass in a 35 mm dish. At 24 h, cells were transfected with variable quantities of pcDNA3.1(−) mouse coproporphyrinogen oxidase (CPO) expression plasmid (gift of Dr Shigeru Taketani (Kohno *et al*, 1993)) using FuGENE 6 transfection reagent (Roche Diagnostics, Indianapolis, IN, USA), exactly as described by the manufacturer. At 24 h post-transfection, the medium was replaced with fresh medium containing 1 mM ALA and incubated for 4 h before analysis of PpIX fluorescence by confocal microscopy. Controls for the analysis included dishes with no treatment, FuGENE only, and ALA only. Fluorescence images along with their corresponding phase-contrast images were captured from regions representing monolayered (not clustered) cells, using a digital camera. Protoporphyrin IX-positive cells were counted from fluorescence images using Adobe Photoshop software, by setting the brightness to a fixed value that rendered approximately half of the cells visible on the ALA-only control slide. The same brightness threshold was then applied to all images and kept constant throughout the analysis. Cells above the threshold were counted for each slide and expressed as the relative number of PpIX-positive cells (% of total cells).

### Cell survival assays

For experiments to measure the effects of MTX upon phototoxicity, sets of 35 mm dishes were plated at a cell number of 8 × 10^4^ (control) or 2 × 10^5^ (MTX treatment), and incubated for 24 h. Then, the control or MTX plates received fresh medium alone, or MTX in the medium, respectively. This procedure assured similar cell densities for both sets of cells at the time of photosensitisation, which was confirmed by actual cell counts in parallel dishes. After 72 h in MTX, cells were incubated with 0.3 mM ALA (for most experiments, unless otherwise noted) for 4 h, and then exposed to graduated doses of light delivered by a 514 nm argon laser (Coherent Inc., Santa Clara, CA, USA) for the clonogenic experiments. Dosimetry was performed using a Coherent Lasermate power meter (Coherent Inc., Santa Clara, CA, USA).

For quantifying short-term phototoxicity, 3-(4,5-dimethylthiazol-2-yl)-2,5-diphenyl-2*H*-tetrazolium bromide (MTT) conversion to formazan at 24 h was monitored by absorbance at 560 nm as described earlier (Ortel *et al*, 1998). This assay has been shown to correlate well with other established measures of cytotoxicity such as colony formation (Iinuma *et al*, 1994).

For quantifying long-term survival, a colony formation assay was used. After performing ALA incubation and light exposures as detailed above, LNCaP cells were detached using trypsin/ethylenediaminetetraacetic acid and resuspended in complete medium. Serially diluted suspensions of the photosensitised cells (range 1 : 5–1 : 1375) were plated on 100 mm dishes and incubated for 13 days. Cells were fixed with 0.2% buffered formalin in methanol and stained with 0.1% aqueous crystal violet. Colonies of more than 50 cells were counted under a dissecting microscope.

To assess if the order of MTX and ALA-PDT is important for efficacy of the combined therapy, colony formation was measured in experiments using a reverse order of administration, that is, ALA-PDT given first, followed by MTX treatment. Cells were exposed for 4 h to ALA (0.3 mM) and irradiated with graduated light doses. Duplicate sets of diluted samples (see above) were then plated on 100 mm dishes in medium either with or without MTX. After 72 h, the medium was replaced by fresh medium in all dishes. After incubation for an additional 10 days, colonies were stained and counted as described above.

### RT–PCR analyses

To detect semiquantitative changes in the level of mRNAs for CPO, PPO, FC, and PBGD in LNCaP cells, the following oligonucleotide (oligo) pairs were used. Coproporphyrinogen oxidase: sense, 5′-CGCAGAAAAGTTCTGAAGAC-3′; CPO: antisense, 5′-CCATCGGGCAGTTAGAGGTA-3′; PPO: sense, 5′-CTGGGAGGTTCCTGGTTACA-3′; PPO: antisense, 5′-CAACCTGTGAGCAGTCAGGA-3′; FC: sense, 5′-GATGAATTGTCCCCCAACAC-3′; FC: antisense, 5′-GCTTCCGTCCCACTTGATTA-3′; and PBGD: sense, 5′-AAGAGTGTGGTGGGAACCAG-3′ and PBGD: antisense, 5′-CATTTCTCAGGGTGCAGGAT-3′. Two micrograms of RNA, extracted from cells using Trizol reagent (Invitrogen) exactly as described by the manufacturer, were used for first-strand synthesis, and the following three different oligos were tried: oligo(dT)_20_ (50 *μ*M); random 9-mer primers (14 *μ*M); and individual gene-specific primers (antisense oligos for the three genes mentioned above, 4 *μ*M each). The primers were employed in reactions with SuperScript III H-reverse transcriptase (Invitrogen), exactly as described by the manufacturer. Antisense G3PDH oligo (BD Biosciences, Palo Alto, CA, USA) was used as internal control for gene-specific first-strand synthesis. For polymerase chain reaction (PCR) amplification, cycle conditions were as follows: 94°C 2 min, 94°C 45 s, 65°C 45 s, 72°C 1 min × 25–35 cycles, and 72°C 10 min. G3PDH control amplimer set was used as internal control for all three different reverse transcription–polymerase chain reactions (RT–PCRs). Amplification products were analysed on a 1.5% agarose gel along with molecular size markers.

A murine CPO cDNA sequence (Kohno *et al*, 1993) was amplified with specific PCR primers and cloned into the TA-cloning vector PCRII-TOPO, then transferred via restriction enzymes *Spe*I/*Xho*I followed by ligation into the *Nhe*I/*Xho*I sites of pcDNA3.1(−), both vectors were from Invitrogen Inc. This mCPO construct was used as a standard for the RT–PCR analyses.

### Epitope-targeted antibody for CPO

A rabbit polyclonal antiserum against CPO was generated from a peptide comprising the last 12 amino acids (LEVLRHPKDWVH) of murine CPO, as per the sequence reported by Kohno *et al* (1993). This peptide has 83% homology between mouse and human at the amino-acid level. The peptide was synthesised with an extra N-terminal cysteine for conjugation to Keyhole limpet antigen in the Biopolymers Core facility of the Massachusetts General Hospital, and sent to HTI Bio-Products Inc. (Ramona, CA, USA) for rabbit immunisation, blood collection, and affinity purification of the antiserum. Specificity was confirmed by Western blots of recombinant mCPO protein expressed in a pcDNA3.1(−) vector transfected into cos-7 cells (see Figure 7C).

### Western blot analyses

Cells were lysed in lysis buffer (7 M urea, 2% IGEPAL, 5% *β*-mercaptoethanol). Samples containing equal quantity of protein, as determined by Bradford's method (Bio-Rad, Hercules, CA, USA), were denatured in sample buffer (Invitrogen) for 10 min at 70°C and resolved on a 4–12% Bis–Tris acrylamide gel (Invitrogen) along with molecular size markers. Electrophoresis was carried out at constant voltage (200 V) at room temperature. Proteins were electrophoretically transferred to Immobilon polyvinylidene difluoride membrane (Millipore, Bedford, MA, USA) at a constant voltage (100 V) for 1 h at 4°C. Following the transfer, the blot was stained with Ponceau Red-S to check the efficiency of transfer, blocked with 10% non-fat dry milk, incubated with rabbit primary antibodies against CPO (1 : 5000), p27 (Santa Cruz Biotech, Santa Cruz, CA, USA) (1:1000), and E-cadherin (Santa Cruz) (1 : 1000) followed by peroxidase-conjugated goat anti-rabbit IgG (1 : 20,000; Jackson ImmunoResearch, West Grove, PA, USA), and developed using enhanced chemiluminescence reagents (ECL kit, Amersham Biosciences, Piscataway, NJ, USA) as described (Passi *et al*, 2004).

### Statistics

Two-sample *t*-tests were used to compare the amount of PpIX accumulation and cytotoxicity between treated and untreated controls (Figures 3, 4, 6 and 7). In addition, analyses of variance (ANOVA) were performed to determine whether there was an additive *vs* synergistic effect in Figure 4 by testing the interaction term between MTX and ALA-PDT. A *P*-value of 0.05 or less was considered statistically significant.

## RESULTS

### Methotrexate stimulates accumulation of intracellular PpIX while promoting growth arrest, differentiation, and apoptosis in LNCaP cells.

**Figure 1:**
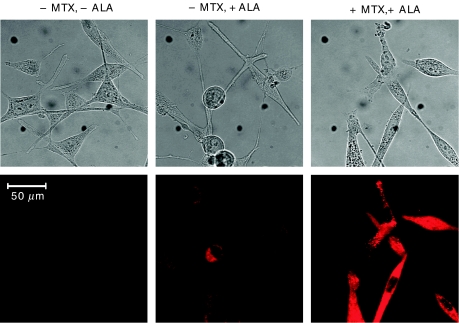
Methotrexate pretreatment stimulates the accumulation of PpIX in LNCaP cells. LNCaP cells, seeded in 35 mm dishes 1 day previously, were incubated in culture medium containing MTX (1 mg l^−1^; +MTX), or in medium without additives (−MTX). After 72 h, ALA was added to some of the dishes (0.3 mM, +ALA; middle and right panels) and the cells were incubated for an additional 4 h. Protoporphyrin IX-specific fluorescence was analysed by fluorescence microscopy (see Materials and Methods). Upper panels: phase-contrast images. Lower panels: PpIX-fluorescent images. Scale bar, 50 *μ*m.

**Figure 2:**
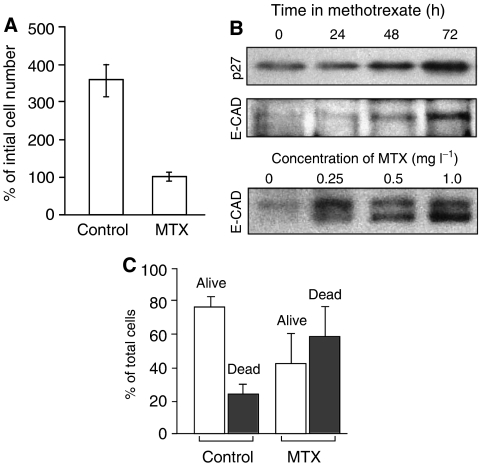
Effect of MTX upon LNCaP growth, differentiation, and apoptosis. (**A**) Inhibition of cell growth by MTX. LNCaP cells were incubated for 72 h in medium containing 1 mg l^−1^ MTX, or solvent alone (control). Proliferation is indicated by a 350% net increase in cell number at 72 h *vs* no increase with MTX. Mean of two experiments carried out in duplicate, ±s.d. (**B**) Western blots of LNCaP cells incubated in the presence of MTX, and analysed with specific antisera to growth arrest marker (p27/kip-1; upper panel) and a differentiation marker (E-cadherin; lower panels). The upper panels show a time course of induction at a fixed MTX concentration (1 mg l^−1^); the lower panel shows a dose–response study with a fixed incubation time of 72 h. The E-cadherin doublet at 124 and 135 kDa (lower panel; Bis–Tris gel) was not well resolved on the smaller, Tris-glycine gel (middle panel). (**C**) Analysis of apoptosis in LNCaP cells after incubation with MTX. Cells were incubated for 72 h in the presence or absence of MTX, as indicated. Adherent and nonadherent cells were pooled and analysed by FACS analysis. The sub-G1 fraction (apoptotic cells, closed bars) and the G1+S+G2/M fractions (living cells, open bars) were expressed as a percentage of total cell number. Bars show mean of six experiments±s.d.

We had previously described a strong increase in PpIX accumulation in epithelial keratinocytes that were differentiating in response to high calcium (Ortel *et al*, 1998), or in LNCaP cells in response to androgens (Ortel *et al*, 2002). To establish whether such a correlation might exist if MTX were used as the prodifferentiating agent, LNCaP cells were pretreated for 72 h with a cytostatic dose of MTX (1 mg l^−1^; see details below), then exposed to ALA for the last 4 h, and analysed for the amount of PpIX synthesised within the cells. Qualitatively, MTX pretreatment led to a significantly higher PpIX accumulation as compared to non-pretreated controls (Figure 1). The same MTX treatment led to growth arrest, as measured by a complete block on cell proliferation (Figure 2A), and by the accumulation of the cell-cycle inhibitor, p27/kip-1 (Figure 2B, upper), whose upregulation correlates with growth arrest in LNCaP cells (Campbell *et al*, 1997). Methotrexate also promoted cellular differentiation, as measured by a significant rise in E-cadherin (Figure 2B, middle), a marker of differentiation in the LNCaP cells (Campbell *et al*, 1997). The induction of the differentiation marker was MTX dose-dependent (Figure 2B, lower). At the 1 mg l^−1^ concentration, MTX also triggered classical apoptosis, as measured by fluorescent-activated cell analysis with annexin V (Figure 2C). The net effect of growth arrest and apoptosis was to reduce the total number of cells in MTX-treated dishes to ∼25% of the number in untreated control dishes at the end of the 72 h period (Figure 2A).

### The mechanism by which MTX enhances accumulation of PpIX involves the haeme synthesis pathway

**Figure 3:**
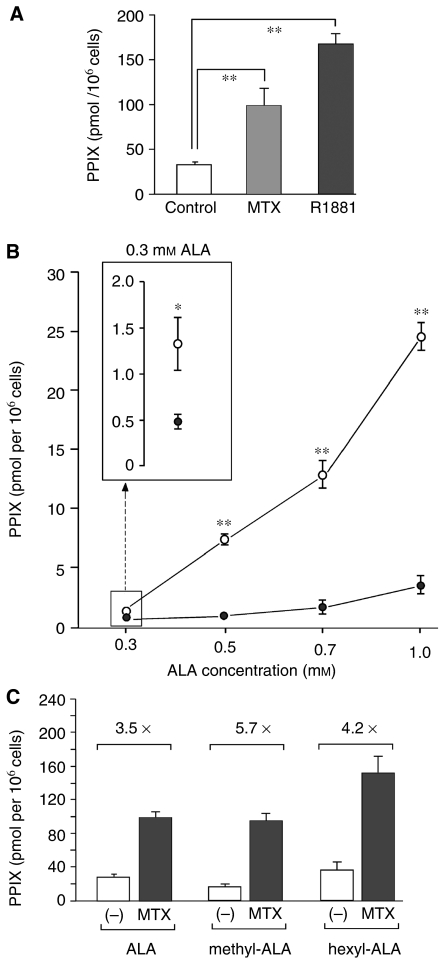
Effect of MTX upon ALA-mediated PpIX accumulation. (**A**) Pretreatment of LNCaP cells with MTX enhances ALA-induced PpIX accumulation. Cells were cultured in the presence of MTX (1 mg l^−1^; grey bar) for 72 h, then ALA (0.3 mM) was added for 4 h, and cells harvested for measurements of intracellular PpIX. For a positive control, some dishes were incubated with an androgen, R1881 (0.1 *μ*M; black bar) for 72 h. Protoporphyrin IX concentrations, calculated from a standard curve, were expressed on a per cell basis. Bars are the mean of eight experiments±s.e.m. (^*^), two-sample *t*-test, *P*<0.001. (**B**) Methotrexate enhances PpIX accumulation in LNCaP cells over a range of ALA concentrations. Cells were pretreated with MTX (open circles) or medium alone (solid circles) for 72 h, then incubated for 4 h in different ALA concentrations before harvest. This experiment shows mean±range of duplicate determinations at each point. Two additional experiments gave qualitatively similar results (not shown). (Inset) Enlargement of the graph at 0.3 mM ALA; note that MTX enhancement of PpIX occurred even at the lowest ALA dose. (Asterisks) Two-sample *t*-test, comparing MTX to control at the same dose of ALA; (^*^) *P*<0.05; (^**^) *P*<0.001. (**C**) Effect of MTX upon accumulation of PpIX derived from different ALA esters. Cells were incubated for 72 h in the absence or presence of MTX, and then for 4 h in the presence of either ALA (0.3 mM), methyl-ALA (0.5 mM), or hexyl-ALA (1 *μ*M) before harvest and PpIX measurement. Bars are the mean±s.e.m. for three experiments, each performed in duplicate. Numbers above each bracket indicate relative increase (-fold) in PpIX owing to MTX. Note the similar -fold increase with all three analogues.

We examined the phenomenon of MTX-mediated enhancement of intracellular PpIX levels in LNCaP cells in detail. Enhancement occurred consistently in eight out of eight experiments, with some variability in magnitude between experiments; typically, a three-fold increase in PpIX was observed in MTX-conditioned *vs* nonconditioned cells (Figure 3A). The PpIX-elevating effect of MTX was only slightly less robust than that of a positive control, the androgen R1881 shown previously to induce PpIX levels in this cell line (Ortel *et al*, 2002).

To study the effect of ALA concentration upon PpIX accumulation, ALA dose-ranging experiments in the presence or absence of MTX were performed. Protoporphyrin IX content was seen to rise monotonically with increasing ALA concentration (Figure 3B), and the slope of the curve was markedly higher in the presence of MTX (open circles) than in its absence (closed circles). Protoporphyrin IX elevation was appreciated even at the lowest ALA concentrations tested (see Figure 3B, inset).

To rule out the possibility that PpIX accumulation in MTX-preconditioned cells might be owing to increased membrane transport of ALA precursors, we used ALA and two of its alkyl esters, 5-methyl-ALA (m-ALA) and 5-hexyl-ALA (h-ALA), in comparative experiments (Figure 3C). Our approach was based on reports that show distinct membrane transport mechanisms for ALA and m-ALA. 5-Aminolaevulinic acid enters cells via membrane pores that also transport *β*-amino acids and GABA, whereas m-ALA uses a different route (Rud *et al*, 2000; Gederaas *et al*, 2001). Although details of the transport mechanism for hexyl-ALA uptake are not known, it also appears to be distinct from *β*-amino-acid transport (Rud *et al*, 2000). To examine whether MTX might preferentially alter PpIX accumulation depending upon whether the prodrug was in the form of free ALA or one of its esters, LNCaP cells were pretreated with MTX or media (control) for 72 h, and then exposed to ALA, m-ALA, or h-ALA for 4 h. Cellular production of PpIX was assessed by quantitative spectrofluorometry (Figure 3C). Because the hexyl ester of ALA is significantly more permeable than ALA itself, it was necessary to use 300 times less h-ALA (1 *μ*M) than ALA (0.3 mM) to achieve a comparable baseline level of PpIX (Figure 3C, fifth *vs* first bar). However, under conditions in which ALA and its esters give similar PpIX levels at baseline, the addition of MTX caused a similar degree of PpIX accumulation (three- to five-fold), regardless of which form of ALA was used. Because it seems highly unlikely that MTX could affect three different membrane ALA-transporting mechanisms to exactly the same extent, the PpIX-elevating action of MTX does not occur primarily through an effect upon precursor uptake.

### Methotrexate enhances the lethal effects of ALA-based photodynamic treatment

**Figure 4:**
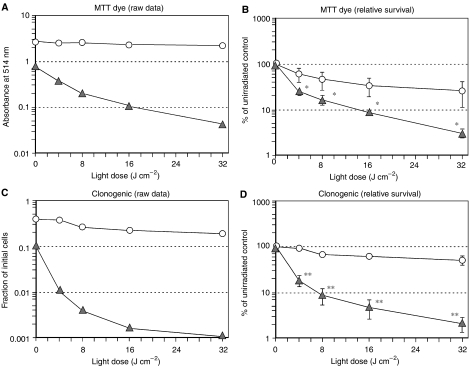
Pretreatment of LNCaP cells with MTX enhances cytotoxicity after the addition of ALA and light. Survival was measured by MTT dye conversion (**A** and **B**) or by colony formation (**C** and **D**), and presented in two ways. Raw data from a single, typical experiment are shown (**A** and **C**), followed by the per cent survival (relative to unirradiated controls), pooled from multiple experiments (**B** and **D**). In all cases, cells were preconditioned for 72 h with MTX (1 mg l^−1^; triangles) or medium alone (circles), then incubated with ALA (0.3 mM) for 4 h and irradiated with light. (**A**) MTT dye assay at 24 h, single experiment. (**B**) MTT dye assay, pooled experiments. Mean±s.e.m. of four experiments. (^*^), *P*<0.05. (**C**) Colony formation assay at 13-day, single experiment. (**D**) Colony formation assay at 13-day, pooled experiments. Mean±s.e.m. of four experiments. (^**^), *P*<0.0005. See Materials and Methods and Results for further details.

We next tested whether the elevated intracellular levels of PpIX, observed in MTX-preconditioned cells, can have functional consequences in terms of cell survival after exposure to visible light. LNCaP cells were pretreated with MTX for 72 h, exposed to ALA for 4 h, and then irradiated with 512 nm light and analysed for cytotoxicity using two different assays, MTT dye conversion (Figure 4A and B), and colony formation (Figure 4C and D). 3-(4,5-Dimethylthiazol-2-yl)-2,5-diphenyl-2*H*-tetrazolium bromide conversion at 24 h is a short-term cytotoxicity assay; MTT serves as an indicator of intact cellular metabolism. In a typical experiment (Figure 4A), MTX caused an initial ∼75% decrease in viability as compared to nonconditioned cells; the subsequent administration of light caused an additional >1 log of cell kill, considerably more than in nonconditioned cells. Because the absolute magnitude of viability data often varies between experiments, we expressed the data as per cent survival (relative to nonirradiated controls) in order to allow the normalisation and pooling of multiple experiments. These aggregate results demonstrated that the enhancement of cytotoxicity in the MTX-preconditioned group was statistically significant (Figure 4B). To assess long-term survival, clonogenic growth of the LNCaP cells at ∼2 weeks after irradiation was plotted against increasing doses of light (Figure 4C). Again, MTX significantly enhanced cell killing in the photodynamic regimen, and when the results of multiple experiments were pooled and compared, the improvement with MTX was highly significant (Figure 4D).

To begin to determine whether MTX and ALA-PDT were working independently to promote cell killing, we subjected the data to an ANOVA. In our experiments, the combination of MTX pretreatment and ALA-PDT were synergistic for cell killing only at the lowest dose of light (4 J cm^−2^, *P*<0.001), but in general the lethal effects of MTX and ALA-PDT were additive.

### The MTX effect upon ALA-mediated cell killing is order-dependent

**Figure 5:**
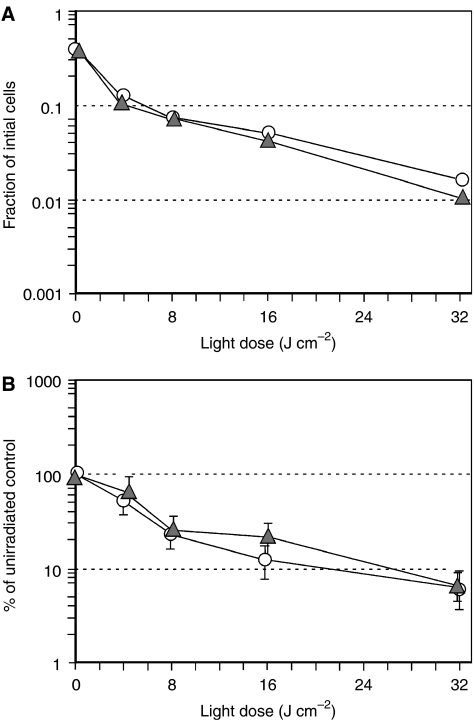
Photodynamic therapy is not enhanced if MTX is administered after ALA and light. Cells were first subjected to ALA-PDT treatment (4 h of ALA 0.3 mM, followed by exposure to graduated light doses) and plated for colony formation assay. Subsequently, one set of plates was exposed to MTX for 72 h, the other to medium only. Colonies were counted at 13 days. (**A**) Colony formation assay, single experiment. (**B**) Colony formation assay, pooled experiments. Mean of four experiments±s.e.m.

Although we hypothesised that enhanced cytotoxicity with MTX occurs because PpIX production is increased in the MTX-preconditioned cells, other possible mechanisms warranted consideration. For example, because MTX inhibits cell proliferation, lower cell densities in the cultures at the time of PDT could hypothetically lead to differences in cellular cytotoxic responses. Therefore, we were careful to eliminate cell density as a variable by adjusting the number of cells plated so as to yield an equivalent cell density at the time of irradiation (see Materials and Methods). An equally important possibility was that MTX might affect cell behaviour after photodynamic treatment, for example, by accelerating cell death pathways. To test this possibility, we reversed the order of MTX and ALA-plus-light treatments (Figure 5). Cells were subjected to ALA and light exposure first, followed by a 72 h exposure to MTX. Under these conditions, no significant differences in long-term cell survival between MTX- *vs* vehicle-treated cells were observed (Figure 5). These data indicate that the presence of MTX does not appreciably alter the mechanisms of cell lethality, once initiated by ALA-PDT.

### Methotrexate at noncytotoxic concentrations induces PpIX levels

**Figure 6:**
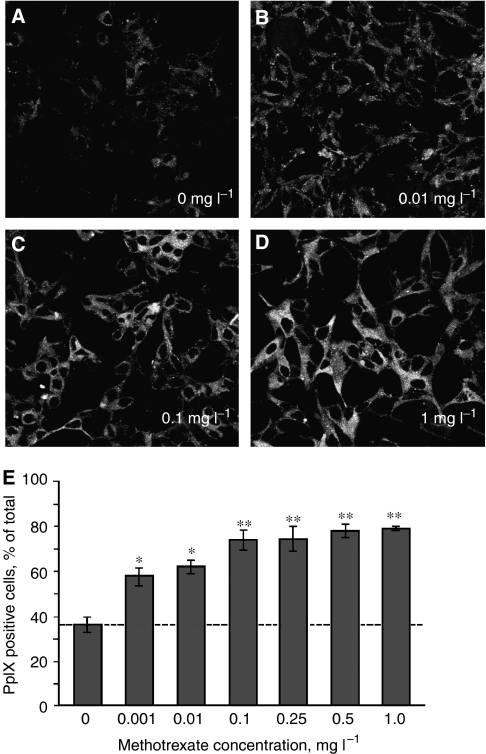
Very low concentrations of MTX can enhance PpIX in LNCaP cells. At time zero, LNCaP cells (3 × 10^5^ cells dish^−1^) were plated on glass cover slips as described in Materials and Methods, and at 24 h, switched to medium containing variable quantities of MTX (0.001–1.0 mg l^−1^). At 72 h, new medium with 1 mM ALA was added and cells were incubated for 4 h before visualisation of PpIX-specific fluorescence by confocal microscopy. (**A**–**D**) Representative images (shown here in grey scale) of PpIX-specific fluorescence in LNCaP cells at the MTX concentrations indicated. (**E**) Semiquantitative analysis of the relative number of cells that were PpIX-positive (% of total cells), performed as described in Materials and Methods. Bars are the mean±s.d. of three digital images per transfection. (^*^) *P*<0.001; (^**^) *P*<0.0005. The experiment was performed twice with similar results.

Whereas the majority of the preceding data were obtained with a relatively high concentration of MTX (1 mg l^−1^=2.2 *μ*M), chosen to approximate the therapeutic levels typically sought in the clinic, MTX at that concentration has antiproliferative and cytotoxic effects in addition to the prodifferentiating effects of particular interest here. To ask whether lower MTX doses might promote accumulation of PpIX, LNCaP cells were exposed to MTX concentrations over a wide range (a 1000-fold, 2.2 *μ*M–2.2 nM) and PpIX levels were assessed by semiquantitative confocal microscopy (Figure 6A–D). The results demonstrated an MTX-dependent increase in PpIX levels, seen with MTX concentrations as low as 2.2 nM (Figure 6E). From this, we conclude that low, nontoxic concentrations of MTX can promote significant accumulation of PpIX.

### Methotrexate enhances PpIX levels via an upregulation of CPO

**Figure 7:**
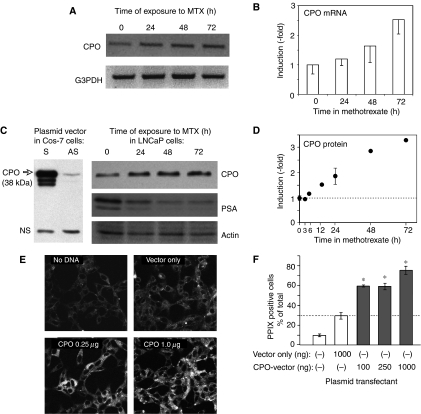
Demonstration that MTX treatment leads to increased levels of CPO. (**A**) Methotrexate treatment of LNCaP cells causes accumulation of CPO mRNA. Cells were exposed for various times to MTX (1 mg l^−1^), or remained untreated (0 h). Cells were lysed, cDNA prepared, and RT–PCR performed using a CPO gene-specific primer set, as described in Materials and Methods. The resulting PCR product was confirmed by DNA sequencing and by size-similarity to a known plasmid cDNA (not shown). The ethidium-stained, CPO-specific bands are shown here inverted (dark bands). G3PDH, invariant control. (**B**) Semiquantitative analysis of CPO mRNA. Agarose gels were digitally photographed, and the integrated density of each band measured using NIH Image software. Induction (relative to untreated controls) was calculated after background subtraction and normalisation to the G3PDH bands. Mean±range of two experiments. (**C**, left side) The anti-CPO epitope antibody recognises full-length CPO protein. Lysates from cos-7 cells were transfected with a plasmid vector (pCPO) expressing CPO inserted in either the sense (S) or antisense (AS) orientation, and analysed by Western blot using the antiserum to CPO. Arrow: CPO-specific signal; NS, nonspecific. (**C**, right side) CPO is expressed in LNCaP cells and increases with MTX treatment. Equal amounts of protein from lysed cells were separated on acrylamide gels, and immunoblotted with antibodies to CPO, prostate-specific antigen, or actin. The 38 kDa CPO protein is indicated. (**D**) Semiquantitative analysis of CPO protein. Western blots from experiments examining long and short times of exposure to MTX were scanned densitometrically, and the data combined. Error bars: range of duplicate experiments. (**E**) Fluorescence images, captured from LNCaP cells that were either not transfected (No DNA), transfected with an empty pcDNA3.1 vector (Vector only), or transfected with different amounts of a plasmid vector overexpressing CPO, followed by incubation with ALA and analysis of the PpIX-specific signal by confocal microscopy. The PpIX-specific signal is shown in grey scale. (**F**) Semiquantitative analysis of relative PpIX-positive cells (% of total cells) was performed as described in Materials and Methods. Bars represent the mean±s.d. of three images from each of two dishes; Asterisks, *P*<0.0005 relative to the vector-only control.

**Figure 8:**
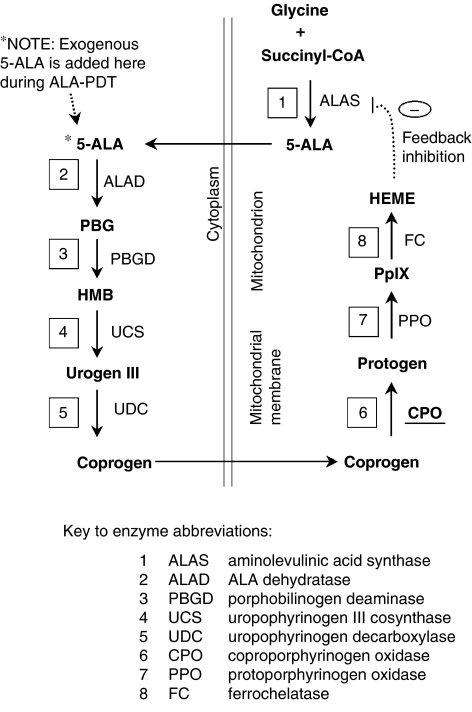
Schematic of porphyrin-synthetic pathway to illustrate potential control points for increased PpIX accumulation. Enzymes, with names abbreviated and relative locations in the pathway shown in boxes, are numbered beginning with the initial condensation of glycine and succinyl-CoA. Substrates/products are shown in bold font. The intracellular location of enzymes, relative to the mitochondria and the cytoplasm, is indicated. See Bickers *et al* (1999) for a fully detailed version.

To test for changes in the enzymes most likely to be involved in an accumulation of PpIX in the haeme synthesis pathway, we considered several potentially rate-limiting enzymes both proximal and distal to PpIX (for a schematic of this pathway, see Figure 8). Given prior evidence that CPO was involved in raising PpIX levels in differentiating epithelial cells (Ortel *et al*, 1998), we were especially interested in whether CPO gene expression was altered by MTX. To test for changes in mRNA expression, LNCaP cells were treated with MTX or with media alone, and RT–PCR was performed. Using gene-specific primers at the reverse transcription step, followed by a CPO-specific primer pair (see Materials and Methods), a significant and reproducible 2.5-fold increase in CPO mRNA level was observed (Figure 7A and B). Reverse transcription–polymerase chain reaction analyses with gene-specific priming for PPO, ferrochelatase, and PBGD demonstrated no changes in mRNA expression (data not shown), indicating no major effects upon gene expression for those enzymes.

To demonstrate the effect of MTX upon CPO at the protein level, we generated a rabbit antiserum to a defined epitope of the CPO enzyme (see Materials and Methods). The antiserum detected a 38 kDa complex on Western blots of lysates from cos-7 cells transfected with a plasmid vector expressing full-length murine CPO (Figure 7C, left). The largest band at 38 kDa corresponds to the predicted size of mature CPO (Ortel *et al*, 1998), whereas the two smaller forms may represent proteolytic processing or translation occurring from internal ribosome-initiation sites. Time-course experiments, from LNCaP cells harvested at different times in MTX-containing media (Figure 7C, right), revealed significant changes in a band with the same relative migration (38 kDa) as authentic CPO in the cos-7 lysates. Quantitation of CPO expression from several experiments showed a reproducible increase in this band, detectable by 12 h and rising three-fold by 48 h (Figure 7D). We conclude that MTX causes a significant accumulation of CPO at both the mRNA and protein levels.

As another way to investigate the link between induced CPO expression and enhanced PpIX levels, a cDNA plasmid vector expressing murine CPO was transiently transfected into LNCaP cells and PpIX levels were observed by confocal microscopy (Figure 7E and F). Compared to an empty-vector control, the CPO-containing vector caused a dose-dependent increase in PpIX signal within the cells (Figure 7E). This increase was statistically significant (Figure 7F).

## DISCUSSION

Owing to the complex nature of cancer development and progression, it is becoming increasingly clear that no single therapeutic modality is likely to be curative. It is now generally accepted that newer molecular mechanism-based therapeutics, particularly those in combination regimens, are more likely to eradicate malignant disease. In this study, we present a novel combination treatment that utilises cellular differentiation and photodynamic destruction of prostate cancer cells. When administered in the proper order, each modality enhances the other, as evidenced by an improved overall outcome in terms of cell killing.

Our demonstration of a new combination therapy for ALA-PDT is important because it captures the advantages of PDT-based localised treatment, while using cellular physiology to produce an overall enhanced outcome. 5-Aminolaevulinic acid-PDT provides the targeting specificity. 5-Aminolaevulinic acid, an inert pharmacological precursor, remains inactive until it is converted into a photosensitising agent (PpIX) within the target tissue, thereby confining phototoxic chemical events to the target cells and reducing toxic side effects (Kennedy *et al*, 1990; Iinuma *et al*, 1994). However, because tumour eradication with ALA-PDT is not always obtained, a new combination that enhances ALA-PDT would be very useful. Four parameters are necessary for effective PDT: the photosensitising agent, light, oxygen, and cellular physiology. Although many laboratories are attempting to optimise the first three (physical) parameters, we are particularly interested in the cell-physiologic aspects. If higher levels of PpIX could be obtained by proper manipulation of the cellular metabolic state, then photodynamic efficacy could be improved. Methotrexate was chosen as an attractive candidate agent because MTX is already used widely in the clinical arena, has relatively low toxicity, and exerts its own antitumour effects by inhibiting proliferation, promoting apoptosis, and promoting terminal differentiation (Hatse *et al*, 1999). The ability to promote differentiation, in particular, suggested to us the idea of using MTX in combination with ALA-PDT. Two other agents (androgens and vitamin D) are known to promote cellular differentiation and at the same time to enhance PpIX accumulation in LNCaP cells (Ortel *et al*, 2002). We decided to test MTX for a similar capability. The concept is to administer MTX in a transient way, preparing the cells for PDT by promoting accumulation of the PpIX photosensitiser.

Our results show that this combined approach is indeed feasible. Preconditioning LNCaP cells with MTX sensitises them to subsequent ALA-PDT, and increases cell killing. An important finding was the fact that for MTX to affect cell lethality, it must be administered before the ALA and light, and not in the reverse order (Figure 5). This suggests that MTX prepares the cell for death by enhancing accumulation of PpIX rather than by affecting postirradiation events. Methotrexate-related increases in PpIX accumulation are substantial (always over three-fold) and nearly as robust as PpIX induction seen with androgenic hormones. The PpIX-elevating effects of MTX occurs across a range of ALA concentrations (Figure 3B), suggesting that despite variations in ALA concentration within tissue, sufficient PpIX might accumulate in various parts of a tumour to provide effective photosensitisation. Further, we found that PpIX elevation occurs over a wide range of MTX concentrations. Very low concentrations of MTX, 100- to 1000-fold lower than the high-dose regimen of 1.0 mg l^−1^ used in the cell-survival portion of our study, can significantly boost the accumulation PpIX in LNCaP cells (Figure 6). This implies that even when MTX penetration into a tumour is relatively poor, a significant enhancement of PpIX may still be possible. The cell-survival studies performed here at MTX 1.0 mg l^−1^ showed a combined effect of MTX and ALA-PDT that was only additive, but this may relate to the very real possibility that the dose of MTX that we were using was (in retrospect) too high to detect a synergistic effect. Further studies are planned to ask whether very low-dose MTX combined with ALA-PDT can produce synergistic killing, as well as to validate whether the phenomenon can be reproduced in three-dimensional tissue culture and animal models.

To investigate the mechanisms of increased PpIX accumulation, we first asked whether MTX might be affecting cellular uptake of 5-ALA. Using different ALA esters as the exogenous source for the production of PpIX (Figure 3C), we found that MTX exerted a similar PpIX-elevating effect, regardless of which ester-specific transport pathway was employed. Although not completely ruling out an effect of MTX upon ALA uptake, the data led us to search for other possible effects of MTX, such as altered PpIX synthesis and/or degradation. For this, we looked at the expression of enzymes in the haeme-synthetic pathway (see cartoon in Figure 8). No changes in mRNA expression were seen for PBGD, PPO, or ferrochelatase. However, a significant three-fold increase in the level of CPO was found to occur. To be sure of these results, we devoted considerable time toward developing an RT–PCR assay that could reliably detect small changes in CPO message levels. Pilot experiments with conventional primers for reverse transcription (oligo-dT, or random hexamers) failed to show any changes in CPO expression, despite amplification in the linear range. Potential problems can occur with the oligo-dT approach when reverse transcription starting at the 3′ end of the RNA leads to under-representation of the 5′ ends of rare transcripts, and random hexamer primers may also amplify an incorrect proportion of transcripts because of failure to produce full-length cDNAs. To resolve these uncertainties, we devised CPO gene-specific primers to direct first-strand cDNA synthesis. Under these conditions, reproducible increases in CPO mRNA from MTX-treated cells were demonstrated (Figure 7B). To show a corresponding increase in CPO protein, it was necessary to develop our own epitope-specific antiserum to CPO. After verifying its specificity against a recombinant CPO protein expressed in cos-7 cells, we used the antiserum to show that LNCaP cells produce CPO at higher amounts in cells preconditioned with MTX (Figure 7C and D). Relative increases in CPO mRNA and protein were modest, ∼three-fold; yet, they can fully account for the three-fold increase in PpIX levels observed in our study. Transfection studies, in which the CPO enzyme was forcibly expressed in the LNCaP cells, confirmed that an elevation in PpIX levels can result from increased expression of CPO (Figure 7E and F).

The idea of a link between terminal differentiation and the CPO enzyme, in the regulation of intracellular porphyrin levels, has some precedence in the literature. In blood, where regulation of porphyrins has been intensively studied, a relationship between CPO levels and erythroid cell differentiation was convincingly established. For example, murine erythroleukaemia MEL cells induced to differentiate by dimethylsulphoxide (DMSO), showed a large induction in mRNA for CPO within 10 h (Kohno *et al*, 1993). Likewise, human erythroleukaemia K562 cells undergoing differentiation in the presence of TGF-*β*, also displayed increased CPO expression (Taketani *et al*, 2001). In erythroid cells, differentiation-inducing agents induced several of the haeme-synthetic enzymes simultaneously (Taketani *et al*, 1995), but even under such circumstances, CPO may be rate-limiting and therefore important in the regulation of haeme and PpIX production (Conder *et al*, 1991). Our own demonstrations of CPO upregulation in differentiating keratinocytes (Ortel *et al*, 1998) and LNCaP cells (Ortel *et al*, 2002) were among the first to demonstrate such a link in nonhaematopoietic cells. How MTX causes an increase in CPO expression in these situations remains an open question, but possibilities for transcriptional regulation of the CPO promoter are intriguing. For example, MTX regulates expression of three transcription factors, Cdx2, GATA-4, and HNF-1*α*, that are important for epithelium differentiation in a mouse model of induced intestinal damage (de Koning *et al*, 2006). Putative binding sites for the above-mentioned transcription factors on the murine CPO promoter might account for increased CPO expression following MTX treatment of LNCaP cells (Takahashi *et al*, 1998; Maytin *et al*, 2006).

The notion that MTX-induced effects upon PpIX levels in the LNCaP cells might be linked to induction of a differentiated phenotype *per se* remains open to conjecture. We have not yet exhaustively investigated the relative effects of very low-dose MTX upon differentiation, apoptosis, and growth arrest of LNCaP (although apoptosis appears to be an insignificant fraction). In human choriocarcinoma (BeWo) cells, a well-established model of MTX-induced differentiation (Friedman and Skehan, 1979); treatment with MTX induces cytodifferentiation at concentrations between 10^−8^ and 10^−7^ M (Burres and Cass, 1987). Similarly in cultured human keratinocytes, MTX in the 10^−8^ to 10^−^7 M range was reported to induce only 1% apoptotic cells (Heenen *et al*, 1998) while inducing significant numbers of differentiated (involucrin-positive) cells (Schwartz *et al*, 1992) in the cultures. In LNCaP cells, at this point, we can only say that MTX (at low nontoxic concentrations) clearly promotes the accumulation of PpIX, and that further studies are underway.

The current study broadens the number of situations in which epithelial-type cancer cells can be manipulated to produce higher levels of PpIX endogenously, in this case using an agent (MTX) commonly used in the clinic for cancer chemotherapy. Future studies will be needed to establish whether MTX can be effectively combined with ALA-PDT in the clinical setting.
